# How does HPV vaccination status relate to risk perceptions and intention to participate in cervical screening? a survey study

**DOI:** 10.1186/s12889-016-3397-y

**Published:** 2016-08-03

**Authors:** Mie Sara Hestbech, Dorte Gyrd-Hansen, Jakob Kragstrup, Volkert Siersma, John Brodersen

**Affiliations:** 1 Section of General Practice and Research Unit for General Practice, Department of Public Health, University of Copenhagen, Øster Farimagsgade 5, PO Box 2099, 1014 Copenhagen K, Denmark; 2 COHERE, Department of Business and Economics & Department of Public Health, University of Southern Denmark, Campusvej 55, 5230 Odense M, Denmark; 3 Primary Health Care Research Unit, Zealand Region, Denmark

**Keywords:** Cervical screening, HPV vaccination, Medical screening, Risk perception

## Abstract

**Background:**

Women in several countries will soon be covered by two preventive programmes targeting cervical cancer: HPV vaccination and cervical screening. The HPV vaccines are expected to prevent approximately 70 % of cervical cancers. It has been speculated, that HPV vaccinated women will not attend screening because they falsely think that the vaccine has eliminated their cervical cancer risk. The aim of this study was to investigate the association between HPV vaccination status and perceptions of cervical cancer risk; perceptions of vaccine effect; and intention to participate in cervical screening. Furthermore, to investigate associations between perceptions of cervical cancer risk and intention to participate in cervical screening.

**Methods:**

A random sample of Danish women from the birth cohorts 1993–1995 was invited to complete a web-based questionnaire concerning risk perceptions and intentions to participate in cervical screening. Main outcomes were: perceived lifetime-risk of cervical cancer; perceived HPV vaccine effect; and intention to participate in cervical screening.

**Results:**

HPV vaccinated women more often than unvaccinated women intended to participate in screening: adjusted odds ratio (OR) for being HPV vaccinated when intending to participate in screening of 3.89 (95 % CI: 2.50–6.06). HPV vaccinated women perceived cervical cancer risk to be higher than unvaccinated women did: adjusted OR of 0.11 (95 % CI: 0.03–0.39) and 0.51 (95 % CI: 0.33–0.78) for being HPV vaccinated while having the lowest perception of risk (in two different pre-specified dichotomisations). HPV vaccinated women perceived the vaccine effect to be larger than unvaccinated women did: adjusted OR of 0.31 (95 % CI: 0.18–0.51) and 0.37 (95 % CI: 0.25–0.53) for being HPV vaccinated while having the lowest perception of vaccine effect (in two different pre-specified dichotomisations). There were no associations between perceived cervical cancer risk and intention to participate in screening.

**Conclusions:**

HPV vaccinated women more often than unvaccinated women intended to participate in screening and they perceived cervical cancer risk to be higher and the vaccine effect to be larger than unvaccinated women did. However, in our analyses, risk perceptions could not explain screening intentions neither among vaccinated nor among unvaccinated women.

**Electronic supplementary material:**

The online version of this article (doi:10.1186/s12889-016-3397-y) contains supplementary material, which is available to authorized users.

## Background

Women in several countries will soon be covered by two preventive programmes targeting cervical cancer: HPV vaccination and cervical screening. Screening for cervical cancer has been followed by a reduction in cervical cancer incidence and mortality assumedly caused by screening [1]. The two HPV vaccines currently on the market prevent infection with HPV types 16 and 18, which are responsible for approximately 70 % of cervical cancers [2]. Thus, a significant reduction in cervical cancer incidence is expected among the women vaccinated before sexual debut. The first women vaccinated as adolescents have recently entered the cervical screening programme, and some countries have revised their screening programmes [3, 4].

Women’s knowledge about cervical cancer is generally poor, and they overestimate their risk of cervical cancer and the effect of cervical screening [5–7]. After implementation of the vaccine, women’s knowledge and attitudes to cervical screening have been investigated [8–13]. The concern has been raised, that the HPV vaccine gives “a false sense of security” because women believe that they have eliminated their risk of cervical cancer [11, 14]. However, these studies were conducted a considerable time before the study populations were invited for screening. A few studies have investigated actual screening behaviour among the first women vaccinated in catch-up programmes: An Australian study suggests that vaccinated women are being screened at lower rates than unvaccinated women [15], while a study from the UK indicates the opposite [16]. However, we still do not know how screening behaviour will be among women vaccinated as adolescents.

Therefore, our aim was to investigate if there was an association between HPV vaccination status and perceptions of cervical cancer risk; perceptions of vaccine effect; and intention to participate in cervical screening. Furthermore, we investigated associations between risk perceptions and intention to participate in cervical screening.

## Methods

### Setting

This study was carried out in Denmark. The Danish Health Authority recommends cytology screening triennially for women aged 23–49 years, and every 5 years in the age group 50–64 [17]. Screening samples are taken by GPs. From 1 October 2008, women born 1993–1995 were offered free HPV vaccination in a catch-up programme, and from 1 January 2009, free vaccination was offered to all girls turning 12 years as a part of the national childhood immunisation programme. Approximately, 80 % of these birth cohorts have received all doses of the vaccine [18], and will enter the cervical screening programme from 2016 and onwards.

### Population and procedure

The study population was females from the birth cohorts 1993, 1994 and 1995. A random sample of these cohorts was drawn using the public register Statistics Denmark. We received personal contact information drawn from public registers. Telephone numbers were matched on names and address. Those who did not have an obtainable publicly registered telephone number were excluded. The remaining potential respondents were sent an SMS (short text message) containing a link to the survey. No gifts, prices or other incentives were used. Data collection started 8 April 2015. Reminders were sent 8 days and 14 days after data collection started. The data collection ended 20 May 2015, when no further responses had been received for more than two weeks.

Survey data were supplemented with socio-demographic data obtained from Statistics Denmark. The Danish Civil Registration System (CRS) contains information on all individuals with a permanent address in Denmark [19]. Each individual receives a unique personal identification number (CPR-number). CPR-numbers were used to link each potential respondent to data from registers in Statistics Denmark. Anonymity was assured by automatically generated ID-numbers replacing CPR-numbers in the analysis database.

We included the following socio-demographic register-based variables: Ethnicity, degree of urbanisation, completed educational level, parents’ educational level, and primary care contacts within the previous year (a measure of use of healthcare services). In order to obtain current information on the women’s educational level, we asked respondents about their on-going education in the questionnaire.

### Questionnaire

A web-based questionnaire, compatible with smartphones and tablets, was developed with the programme Survey Xact.

In the first part of the questionnaire, respondents were asked about their educational level and HPV vaccination status. Subsequently, they were asked about their perception of lifetime-risk of cervical cancer for an *HPV unvaccinated* woman and successively for an *HPV vaccinated* woman. In the second part of the questionnaire all potential respondents were randomised to one of four study arms receiving four different information modules, respectively. Finally, all respondents were asked about their intention to participate in cervical screening. The questionnaire did not allow respondents to go back and change their responses to previous questions, thus the questions concerning risk perceptions were not affected by the information modules later in the questionnaire.

The results from the randomised trial of the effect of the information modules will be reported elsewhere [20]. In the present study, we focused on data concerning vaccination status and risk perception collected prior to randomisation to the information modules, and on intention to participate in screening assessed after the information modules. We used data from all four study arms. There were no statistically significant socio-demographic differences between study arms [20].

The first draft of questionnaire items was made through an iterative process involving consecutive discussions among all authors. The final version was generated via qualitative interviews with 12 women in the target group. A combination of two techniques was applied: *Think-aloud interviewing* and *cognitive probing* [21, 22], ensuring functionality, understandability and content validity (ad-hoc translated version of the questionnaire is available as Additional file 1).

### Outcomes

We used four self-reported outcomes: Perceived lifetime-risk of cervical cancer conditional on negative HPV vaccination status; perceived lifetime-risk of cervical cancer conditional on positive HPV vaccination status; perceived HPV vaccine effect; and intention to participate in cervical screening.

We asked respondents to report their perceptions of i) lifetime-risk of cervical cancer for an *HPV unvaccinated* woman (*unvaccinated cervical cancer risk*) and ii) lifetime-risk of cervical cancer for an *HPV vaccinated* woman (*vaccinated cervical cancer risk*). Respondents were to provide their answer in terms of number of women per 1000 women that will develop cervical cancer before the age of 75. The answer was to be given on a slider ranging from zero to 1000.

In our subsequent analyses, we calculated each respondent’s perception of the risk reduction in percentage attained by the HPV vaccine by dividing their estimate of *vaccinated cervical cancer risk* with their estimate of *unvaccinated cervical cancer risk* multiplied with 100. Thus, we did not ask respondents directly about their perception of the vaccine effect. The rationale was to make the questionnaire as short and as easy to understand as possible. Furthermore, we wanted an intuitive answer; we did not expect the majority of the respondents to have the exact answer to this question.

In our analyses of association between risk perceptions and intention to participate in screening, we matched vaccination status with the relevant perceived cervical cancer risk.

The distributions of the risk perception outcomes differed between HPV vaccinated and unvaccinated respondents (Figs. 2, 3 and 4). For both groups distributions were non-normal with extreme outliers. Therefore, for robust analyses that were not dependent on the comparison of means or other measures of centrality, we used logistic regression on dichotomised versions of the risk perception outcomes defined by different cut-off values. Perceived cervical cancer risk was dichotomised by three cut-offs: <11 per 1000 vs. ≥11 per 1000; <101 per 1000 vs. ≥101 per 1000; and <501 per 1000 vs. ≥501 per 1000. The first cut-off was based on the actual lifetime-risk in Denmark, which is 9 cervical cancer cases per 1000 women [23]. The two other cut-offs (101 and 501) were based on the graphical distribution of responses, dividing responses in groups that matched the range of the responses (cut-offs are marked in Figs. 2 and 3).

Perceived HPV vaccination effect was likewise dichotomised by three cut-offs: ≤0 % vs. >0 %; <60 % vs. ≥60 %; and <95 % vs. ≥95 %. The first cut-off grouped the cases where respondents reported a higher risk for a vaccinated woman than for an unvaccinated woman, producing a negative vaccination effect (indicating miscomprehension). The second cut-off was set at the lower limit of an approximately correct estimate of the expected vaccine efficacy which is approximately 70 % [2]. The third cut-off grouped those respondents who perceived the vaccine effect to be nearly complete (cut-offs are marked in Fig. 4).

Intention to participate in cervical screening is an accepted outcome measure, previously used in the context of cervical screening [24]. The question “Do you think that you will attend cervical screening?” had three response categories: “Yes”, “no” and “I do not know”. We dichotomised the outcome by merging “no” and “I do not know”.

### Statistical analyses

Analyses of the relations between (i) HPV vaccination status and intention to participate in screening (ii) HPV vaccination status and risk perceptions and (iii) risk perceptions and intention to participate in screening were conducted applying logistic regression analysis. We conducted the unadjusted analyses as well as adjusted for the following socio-demographic variables: Year of birth, ethnicity, degree of urbanisation, completed educational level, self-reported on-going educational level, parents’ educational level, and primary care contacts within the previous year. Moreover, we adjusted for study arm including interaction between study arm and HPV vaccination status (thereby controlling for the information provided in the randomised trial previously described). All calculations were made in SAS v9.4.

## Results

### Participant flow and recruitment

**Fig. 1:**
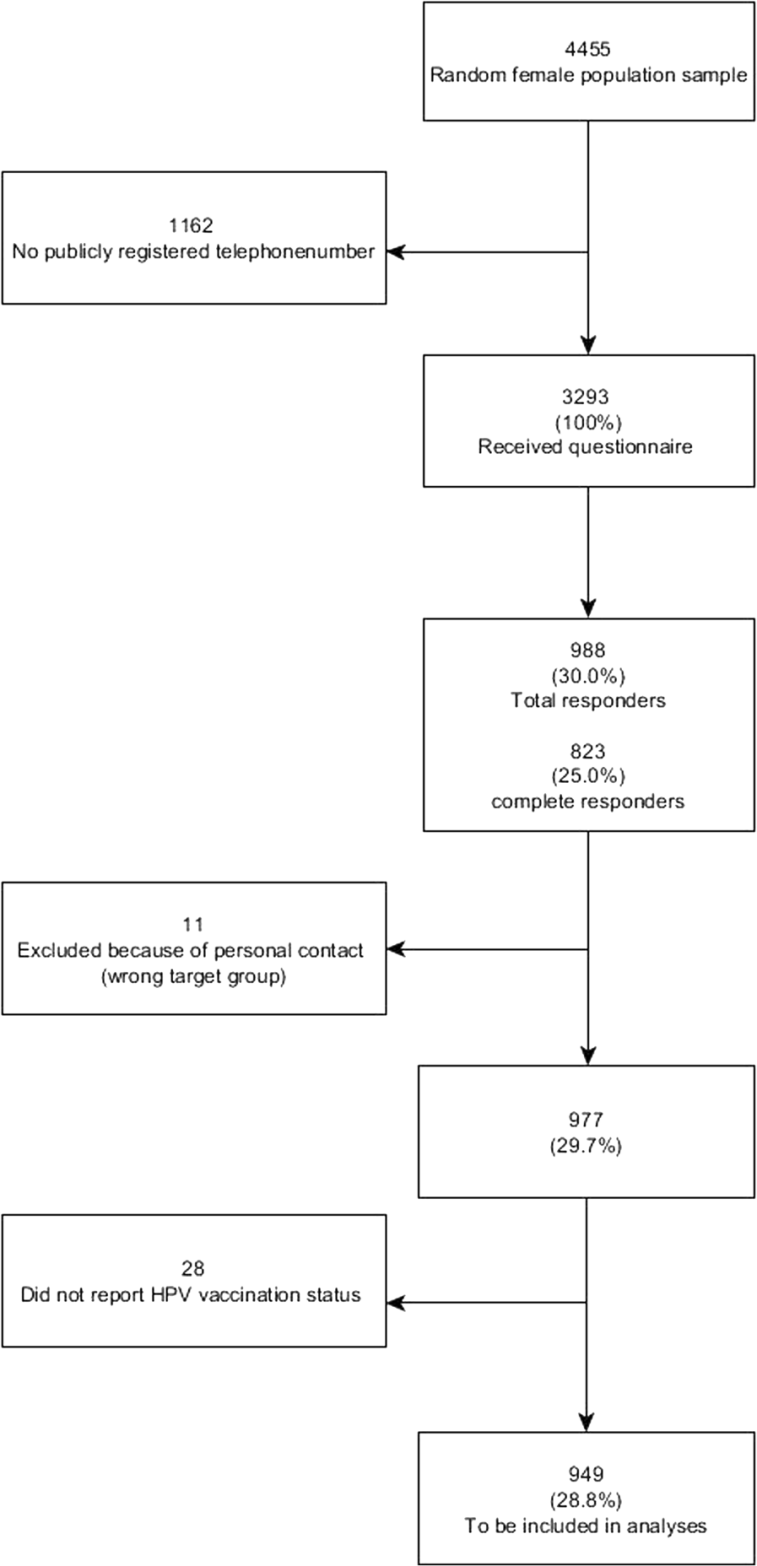
Flow-diagram of participants

In the random sample of 4455 women, 3293 (73.9 %) had a publicly registered telephone number. The overall response rate was 30.0 % (*n* = 988) with 25 % (*n* = 823) completing all parts of the questionnaire. We received 26 e-mails from potential respondents, who had received an SMS but were not in the target group (males, older age group). Eleven of these 26 persons had responded to the questionnaire and their responses were excluded from the analyses. Of the remaining 977, 949 (28.8 %) completed the question concerning HPV vaccination status and were included in the analyses (Fig. 1).

### Socio-demographic characteristics and drop-out analyses

Socio-demographic characteristics of the total study sample are listed in Table 1. Drop-out analyses revealed differences between women with no available telephone number, non-respondents and respondents on all included variables. Relative to non-respondents, respondents were more frequently ethnic Danes and more likely to have more than primary education (+9 years of schooling), as were their parents. They also more often lived in a densely populated area and had more primary-care contacts in the previous year.

**Table 1:** Baseline characteristics and drop-out analyses

	Respondents	Non-respondents	Telephone number unavailable^a^	Total	p-value	Total n in each group included in analysis
	n (%)	n (%)	n (%)	n (%)		(n/n/n)
Total n in population	977	2305	1173	4455		
Year of birth						977/2305/1173
1993	349 (40.58)	658 (28.55)	349 (35.72)	1483 (33.29)		
1994	320 (31.97)	788 (34.19)	320 (32.75)	1483 (33.29)		
1995	308 (27.45)	859 (37.27)	308 (31.53)	1489 (22.42)	<0.0001	
Etnicity						954/2249/1078
Danish	879 (92.14)	1974 (87.77)	924 (85.71)	3777 (88.23)		
Other	75 (7.86)	275 (12.23)	154 (14.29)	504 (11.77)	<0.0001	
Urbanisation						977/2305/1173
Dense	224 (22.93)	370 (16.05)	290 (24.72)	884 (19.84)		
Intermediate	102 (10.44)	282 (12.23)	144 (12.28)	528 (11.85)		
Thinly pop.	651 (66.63)	1653 (71.71)	739 (63.00)	3043 (68.31)	<0.0001	
Educational level (completed)^b^						931/2196/1036
I + II + III + IV	359 (38.56)	633 (28.76)	432 (41.70)	1424 (34.17)		
V	572 (61.44)	1563 (71.24)	604 (58.30)	2739 (65.83)	<0.0001	
Parents educational level^bd^						930/2173/1022
I	37 (3.98)	88 (4.05)	31 (3.03)	156 (3.78)		
II	118 (12.69)	230 (10.58)	114 (11.15)	462 (11.20)		
III	35 (3.76)	79 (3.64)	38 (3.72)	152 (3.68)		
IV	477 (51.29)	1044 (48.04)	455 (44.52)	1976 (47.90)		
V	263 (28.28)	732 (33.69)	384 (37.57)	4125 (33.43)	0.0050	
Number of primary care contacts in the previous year^c^						977/2305/1173
0	83 (8.50)	228 (9.89)	204 (17.39)	515 (11.56)		
1–10	497 (50.87)	1133 (49.15)	508 (43.31)	2138 (47.99)		
11–20	221 (22.62)	532 (23.08)	255 (21.74)	1008 (22.63)		
>20	176 (18.01)	412 (17.87)	206 (17.56)	794 (17.82)	<0.0001	

^a^ Including 11 responders that were outside target group

^b^ Classified according to highest completed (or on-going) level of education: 1: Second stage of tertiary education, 2: First stage of tertiary education, bachelors and equivalent, 3: Post-secondary non-tertiary education, 4: Secondary education, 5: Primary education

^c^ Personal contacts for all purposes, telephone- and e-mail contacts

^d^The highest level of mother or father

Socio-demographic characteristics of HPV vaccinated respondents versus unvaccinated respondents are listed in Table 2. Statistically significant differences were found between these two groups: HPV vaccinated respondents were more frequently of Danish ethnicity and more often living in a densely populated area than unvaccinated. Women with only primary education were over-represented among the unvaccinated.

**Table 2:** Baseline characteristics by vaccination status

	HPV vaccinated respondents	Non-vaccinated respondents	Total	p-value	Total n in each group included in analysis
	n (%)	n (%)	N (%)		(n/n)
Total n in population	698	251	949		
Year of birth					698/251
1993	270 (38.68)	69 (27.49)	339 (35.72)		
1994	236 (33.81)	78 (31.08)	314 (33.09)		
1995	192 (27.51)	104 (41.43)	296 (31.19)	0.0001	
Etnicity					682/244
Danish	641 (93.99)	212 (86.89)	853 (92.12)		
Other	41 (6.01)	32 (13.11)	73 (7.88)	0.0004	
Urbanisation					698/251
Dense	174 (24.93)	43 (17.13)	217 (22.87)		
Intermediate	77 (11.03)	21 (8.37)	98 (10.33)		
Thinly pop.	447 (64.04)	187 (74.50)	634 (66.81)	0.0100	
Educational level (completed)^a^					669/236
I + II + III + IV	288 (43.05)	64 (27.12)	352 (38.90)		
V	381 (56.95)	172 (72.88)	553 (61.10)	<0.0001	
Self-reported educational level (on-going)^a^					698/251
I	126 (10.05)	25 (9.96)	151 (15.91)		
II	109 (15.62)	49 (19.52)	158 (16.65)		
III	37 (5.30)	8 (3.19)	444 (46.79)		
IV	366 (52.44)	78 (31.08)	114 (12.01)		
V	38 (5.44)	76 (30.28)	37 (3.90)		
Unknown	22 (3.15)	15 (5.98)	37 (3.90)	<0.0001	
Parents educational level^ac^					676/228
I	28 (4.14)	8 (3.51)	36 (3.98)		
II	83 (12.28)	30 (13.16)	113 (12.50)		
III	24 (3.55)	11 (4.82)	35 (3.87)		
IV	358 (52.96)	106 (46.49)	464 (51.33)		
V	183 (27.07)	73 (32.02)	256 (28.32)	0.4260	
Number of primary care contacts in the previous year^b^					698/251
0	54 (7.74)	26 (10.36)	80 (8.43)		
1–10	358 (51.29)	127 (50.60)	485 (51.11)		
11–20	159 (22.78)	56 (22.31)	215 (22.66)		
>20	127 (18.19)	42 (16.73)	169 (17.81)	0.6231	

^a^ Classified according to highest completed (or on-going) level of education: I: Second stage of tertiary education, II: First stage of tertiary education, bachelors and equivalent, III: Post-secondary non-tertiary education, IV: Secondary education, V: Primary education

^b^ Personal contacts for all purposes, telephone- and e-mail contacts

^c^The highest level of mother or father

### Main results

**Fig. 2:**
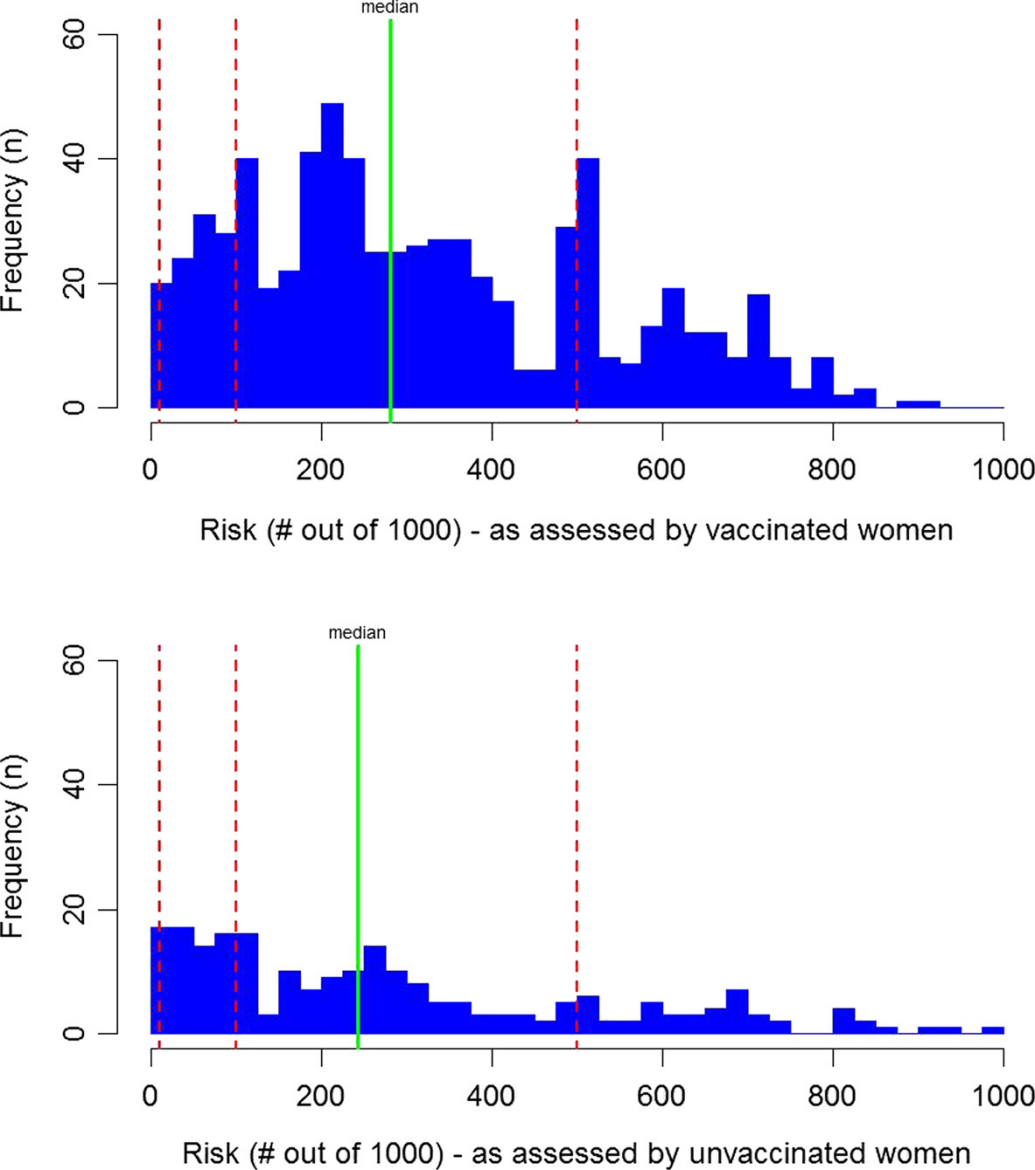
Unvaccinated cervical cancer risk (number of HPV unvaccinated women per 1000 who will develop cervical cancer before the age of 75) as assessed by HPV vaccinated and unvaccinated respondents (x-axis: assessed risk, y-axis: number of respondents) (*Green line*: median. *Dotted red lines*: cut-offs used in analyses)

**Fig. 3:**
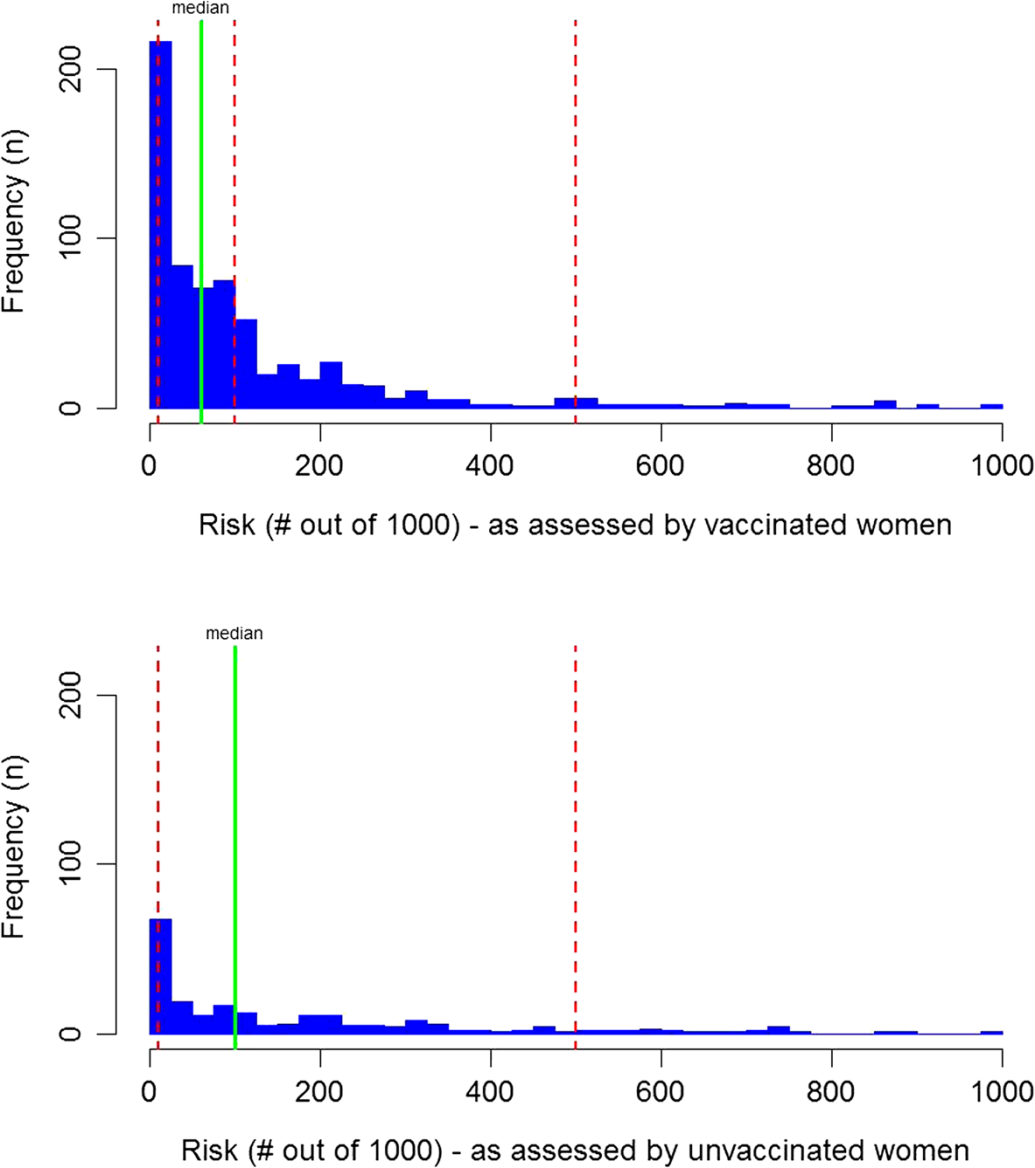
Vaccinated cervical cancer risk (number of HPV vaccinated women per 1000 who will develop cervical cancer before the age of 75) as assessed by HPV vaccinated and unvaccinated respondents (x-axis: assessed risk, y-axis: number of respondents) (*Green line*: median. *Dotted red lines*: cut-offs used in analyses)

**Fig. 4:**
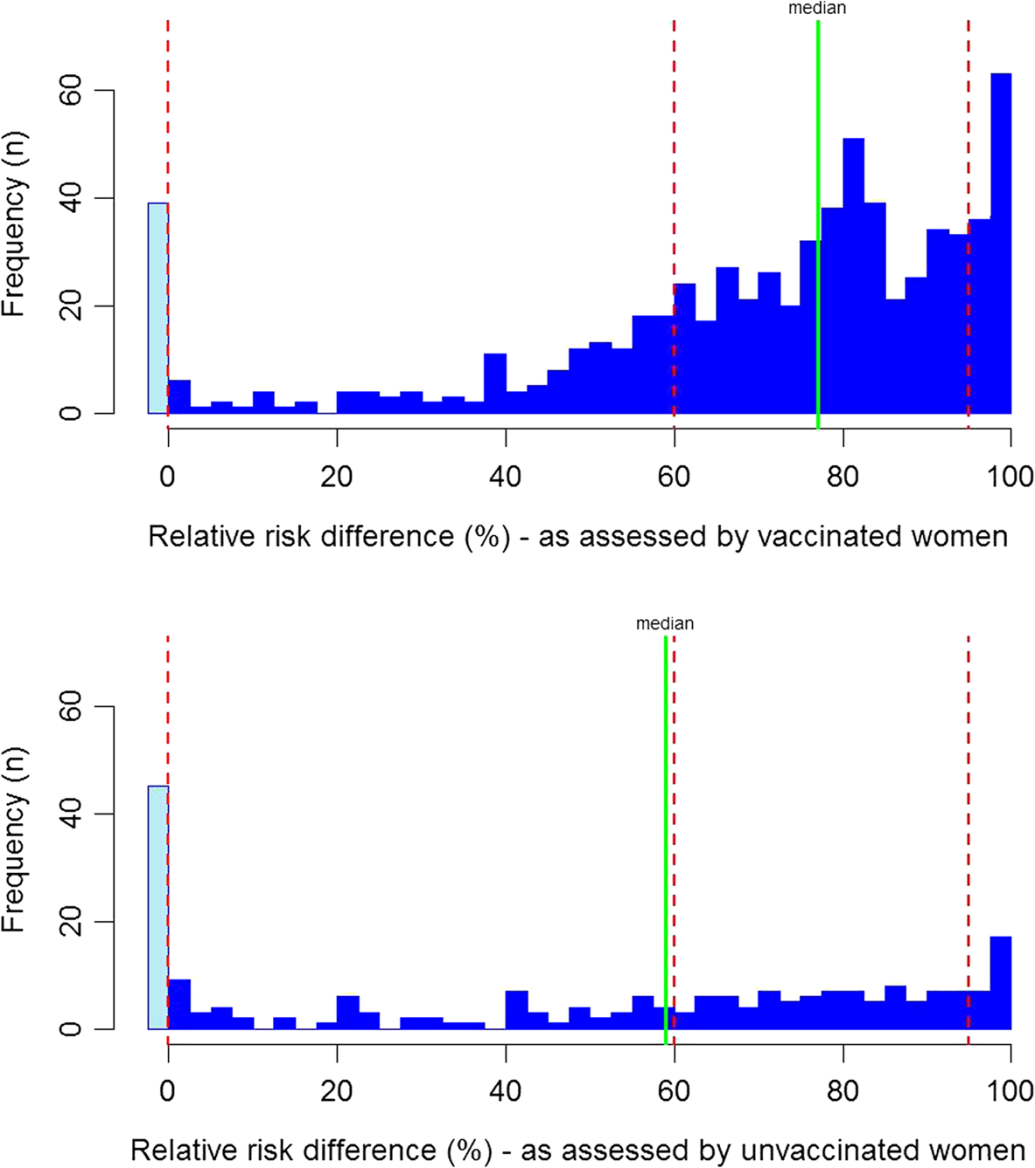
Implied HPV vaccine effect (%) (relative risk difference between *unvaccinated cervical cancer risk* and *vaccinated cervical cancer risk*) as assessed by HPV vaccinated and unvaccinated respondents (x-axis: assessed risk difference, y-axis: number of respondents) (*Green line*: median. *Dotted red lines*: cut-offs used in analyses)

Distributions of i) *unvaccinated cervical cancer risk*, ii) *vaccinated cervical cancer risk,* iii) the implied HPV vaccine effect are shown in Figs. 2, 3 and 4. The median perceived *unvaccinated cervical cancer risk* was 261 per 1000 (282 per 1000 among HPV vaccinated respondents, 242 per 1000 among unvaccinated respondents) (Fig. 2). The median perceived *vaccinated cervical cancer risk* was 68 per 1000 (59 per 1000 among HPV vaccinated respondents, 77 per 1000 among unvaccinated respondents) (Fig. 3). Thus, the median perceived HPV vaccine effect was 74.6 % (77.1 % among HPV vaccinated respondents, 58.9 % among unvaccinated respondents) (Fig. 4). The proportion of HPV vaccinated women who intended to participate in screening was 87.1 and 72.5 % of the unvaccinated women. The adjusted odds ratio (OR) for being HPV vaccinated when intending to participate in screening was 3.89 (95 % CI: 2.50–6.06) (Table 3).

**Table 3:** HPV vaccination status as predictor of screening intention and risk perceptions

Outcome	Total n included in analysis (unadjus-ted/adjusted)	n(%) HPV vaccinated with outcome (unadjusted/adjusted)	n(%) non-vaccinated with outcome (unadjusted/adjusted)	OR (unadjusted) for being HPV vaccinated (95 % CI)	p-value (unadjusted)	OR (adjusted)^a^for being HPV vaccinated (95 % CI)	p-value (adjusted)
Screening intention							
Yes vs. no/do not know	835/783	562 (87.1)/535 (87.0)	119 (62.6)/105 (62.5)	4.04 (2.78–5.87)	<0.0001	3.89 (2.50–6.06)	<0.0001
Perceived cervical cancer risk^b^							
<11 per 1000 women	910/856	5 (0.7)/5 (0.8)	12 (5.4)/11 (5.5)	0.13 (0.05–0.37)	0.0001	0.11 (0.03–0.39)	0.0005
<101 per 1000 women	910/856	103 (15.0)/98 (14.9)	64 (28.6)/55 (27.5)	0.44 (0.31–0.63)	<0.0001	0.51 (0.33–0.78)	0.0019
<501 per 1000 women	910/856	523 (76.2)/500 (76.2)	177 (79.0)/158 (79.0)	0.85 (0.59–1.23)	0.3917	0.71 (0.46–1.09)	0.1207
Perceived HPV vaccine effect							
<0 %	904/851	43 (6.3)/43 (6.6)	53 (24.3)/44 (22.6)	0.21 (0.13–0.32)	<0.0001	0.31 (0.18–0.51)	<0.0001
<60 %	904/851	179 (24.8)/163 (24.9)	109 (50.0)/96 (49.2)	0.33 (0.24–0.45)	<0.0001	0.37 (0.25–0.53)	<0.0001
<95 %	904/851	569 (82.9)/542 (82.6)	191 (87.6)/173 (88.7)	0.69 (0.44–1.08)	0.1024	0.67 (0.39–1.13)	0.1347

^a^Adjusted for study arm and the following socio-demographic variables: Year of birth, ethnicity, degree of urbanisation in area of habitat, completed educational level, parents’ educational level, and primary care contacts within the previous year

^b^ for an unvaccinated woman

We found that HPV vaccinated women perceived cervical cancer risk to be higher than unvaccinated women did. The association between respondents’ own HPV vaccination status and perceptions of *unvaccinated cervical cancer risk* was statistically significant in the two pre-specified dichotomisations with the lowest cut-offs. (Adjusted OR of 0.11 (95 % CI: 0.03–0.39) for being vaccinated while being in the lowest risk perception group when cut-off was set at <11 per 1000 women; adjusted OR of 0.51 (95 % CI: 0.33–0.78) for being vaccinated while being in the lowest risk perception group when cut-off was set at <101 per 1000 women) (Table 3).

We found that HPV vaccinated women perceived the vaccine effect to be larger than unvaccinated women did. The association between respondents’ HPV vaccination status and perceived vaccine effect was statistically significant in the two pre-specified dichotomisations with the lowest cut-offs. (Adjusted OR of 0.31 (95 % CI: 0.18–0.51) for being vaccinated while being in the lowest group when cut-off was set at <0 %; adjusted OR of 0.37 (95 % CI: 0.25–0.53) for being vaccinated while being in the lowest group when cut-off was set at <60 %) (Table 3).

Finally, we found that there were no significant associations between perceived cervical cancer risk subject to HPV vaccination status and intention to participate in screening for none (with one exception) of the pre-specified dichotomisations (see Additional file 2: Table S1). To test whether the association could have been influenced by the information modules, we tested the addition of an interaction between study arm and perceived cervical cancer risk on the outcome intention to participate in screening in the models. None of these interactions were found significant.

## Discussion

HPV vaccinated women more often than unvaccinated women intended to participate in screening and they perceived cervical cancer risk to be higher and the vaccine effect to be larger than unvaccinated women did. However, in our analyses, risk perceptions could not explain screening intentions among neither vaccinated nor unvaccinated women.

The response rate of 29.7 % is low, but the age group might be considered a “hard-to-reach” group in the context of survey research. Drop-out analyses reveal socio-demographic participation bias: respondents seem to come from better educated families. Completing the questionnaire would potentially have been more cognitively challenging for the non-respondents, which might have created increased random error due to incomplete understanding of the questionnaire. We, however, have no reason to believe that our results are highly sensitive to this response bias.

Some respondents implicitly reported a negative effect of the HPV vaccine. We assume that these results are due to misinterpretations of the questions. Since our measure of respondents’ perceptions of the effect of HPV vaccination are derived indirectly from the former two questions on cervical cancer risk subject to HPV status, any misinterpretations of these two questions will spill over onto the perceived HPV effect. The bias introduced by such random errors was reduced by dichotomising risk-perception outcomes.

One may question web-based survey methods and our use of smartphones via an SMS-link as administration form. However, several studies have found that the validity of web-based methods of administration are equal to paper questionnaires [25, 26]. Moreover, studies have shown that the use of PDA, which is highly comparable to a modern smartphone, as administration method is not inferior to other more commonly used methods in survey research [27, 28]. Since the target group of this survey was young women who are generally very familiar with the use of smartphones [29], we believe that our method of administration is a strength of this study. Furthermore, the use of personal telephones potentially increased the likelihood that the intended individual answered the survey. The electronic format does, however, introduce a potential source of bias: Respondents were to provide their answer to the risk perception questions on a slider ranging from 0 to 1000 with the slider set at the default position 500. This might have caused some women to place the slider near the default position. Although we thought to minimise this problem by designing the questionnaire such that one could not proceed without moving the slider, we still observe a “hump” around 500 for the question concerning cancer risk conditional on negative HPV vaccination status (Fig. 2). Since the use of a slider is a relatively new method in survey research, there are, to our knowledge, no studies validating an electronic slider. However, the slider is comparable to a VAS scale, and the hump we observe could also be caused by central tendency bias, which is known from scales in general.

A strength of this study is that we were able to control for a number of potential confounders by adjusting for a number of socio-demographic variables obtained from registers and questionnaire data.

Our study confirms previously observed socio-demographic inequalities between HPV vaccinated and unvaccinated women [16, 30–32]. The proportion of women intending to participate in screening in our study is a little higher than current screening rates in Denmark among vaccinated as well as unvaccinated women [33]. Overall, respondents, HPV vaccinated as well as unvaccinated, had highly inflated perceptions of cervical cancer risk. Such overestimations have been observed previously in other survey studies conducted before and after implementation of the HPV vaccine [5, 9]. However, HPV vaccinated women perceived the risk conditional on negative HPV vaccination status as higher and the risk conditional on positive HPV vaccination status as lower than unvaccinated women did. In other words, HPV vaccinated women perceived the vaccine to be more effective than did unvaccinated women. This could possibly be explained by the classic theory of *cognitive dissonance* [34]: women who are unvaccinated might unconsciously evaluate the HPV vaccine less favourably to resolve any dissonance between their perception of the vaccine and their own vaccination status.

Risk perceptions should expectedly be a predictor of screening intentions. However, we were not able to demonstrate such a relation in this study. This could be due to lack of power, since there was a tendency towards women (vaccinated as well as unvaccinated) with lower perception of cervical cancer risk to be less inclined to participate in screening.

We found a strong association between HPV vaccination status and intention to participate in screening with more HPV vaccinated women than unvaccinated women intending to participate, even after adjusting for a number of socio-demographic variables. This is in line with the findings from a previous survey of Scottish school girls [35] and a cohort study from the UK [16]. Our results suggest that we should not expect that vaccinated women will not attend screening due to a false assumption that they are completely protected against cervical cancer. This is also supported by a median perceived HPV vaccine effect of 77.1 % among HPV vaccinated women, indicating an overall realistic perception of the vaccine effect. That unvaccinated women might choose not to attend cervical screening can, however, raise concern. Socio-demographic inequality is well-known in screening programmes [36–41]. A previous study has specifically shown that non-attenders in the Danish cervical screening programme have less favourable socio-demographic profiles than attenders, e.g. shorter education and limited use of primary health care [42]. If non-attenders are also less likely to be HPV vaccinated than attenders are, inequity in health will be aggravated.

## Conclusions

Our study suggests that HPV vaccinated as well as unvaccinated women have inflated perceptions of cervical cancer risk. Moreover, vaccinated women are much more inclined to participate in screening than unvaccinated women. This is concerning, since those who would benefit the most from screening might not participate, while healthcare resources are spent on those who benefits least. The implications for practice are mainly pertaining to future information interventions directed at women invited for cervical screening. Both groups of women should be provided with clear and balanced information about their personal cervical cancer risk (i.e. according to HPV vaccination status) and the benefits and harms of screening based on best available evidence, so that they have the best possible prerequisites to make an informed choice.

## Additional files

Additional file 1: English translation of questionnaire. (DOCX 112 kb) Additional file 2: Table S1. Perceived cervical cancer risk as predictor of screening intention. (DOCX 14 kb)
