# Supplementary material for: How does HPV vaccination status relate to risk perceptions and intention to participate in cervical screening? a survey study
Source: BMC Public Health. 2016 Aug 3;16:708. doi: 10.1186/s12889-016-3397-y (PMC4973036; doi:10.1186/s12889-016-3397-y)
Supplement: Additional file 1: — English translation of questionnaire. (DOCX 112 kb) [file 12889_2016_3397_MOESM1_ESM.docx]

Questionnaire in full length (ad hoc English translation)

| This questionnaire concerns your knowledge and thoughts about cervical cancer and the HPV vaccine, also known as the cervical cancer vaccine. The HPV vaccine is offered to all young girls in Denmark. HPV (Human Papilloma Virus) is a virus which is passed on by sexual contact. HPV is the most important cause of cervical cancer and genital warts (condyloma). Therefore, the vaccine can prevent cervical cancer and genital warts.  The questionnaire contains some information on this topic. It is important for this survey that you read it thoroughly.  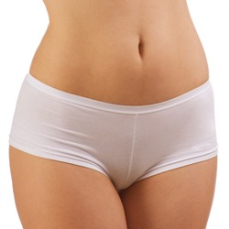 |
| --- |

**Have you completed, or are you currently undertaking secondary education (for example high school or vocational education)?**

- I have completed secondary education
- I am currently undertaking secondary education
- I have not completed/am not currently undertaking secondary education

**What type of secondary education have you completed or are you currently undertaking? (Tick the box at the most recent)**

- High school *(examples of types of Danish high-school educations are given)*
- Vocational education *(examples of types of Danish vocational educations are given)*
- Individual educational course
- I do not know

**Have you completed, or are you currently undertaking tertiary education (for example a bachelor or a masters degree)?**

- I have completed tertiary education
- I am currently undertaking tertiary education
- I have plans to start tertiary education within the next year
- I have not completed/am not currently undertaking tertiary education

**What type of tertiary education have you completed or are you currently undertaking? (Tick the box at the most recent)**

- Short tertiary education of less than 3 years *(examples of Danish short tertiary educations are given)*
- Intermediate tertiary education of 3-4 years *(examples of Danish intermediate length tertiary educations are given)*
- Long tertiary education of more than 4 years *(examples of Danish long tertiary educations are given)*
- Other education
- I do not know

**Have you been vaccinated against HPV (the cervical cancer vaccine)?**

- Yes, all three jabs.
- Yes, two jabs.
- Yes, one jab.
- Yes, do not know how many jabs.
- No
- I do not know

We are now going to ask you some questions concerning cervical cancer risk. Even though you do not know the answer, please try to answer with your best guess.

**How large do you think the risk of getting cervical cancer is for a woman your age, who has not been HPV vaccinated? That is, how many out of 1000 non-vaccinated women will get cervical cancer before the age of 75 years?**

*(The answer has to be given on a slider ranging from 0 to 1000)*

**How large do you think the risk of getting cervical cancer is for a woman your age, who has been HPV vaccinated? That is, how many out of 1000 vaccinated women will get cervical cancer before the age of 75 years?**

*(The answer has to be given on a slider ranging from 0 to 1000)*

**When you continue completing the questionnaire, you will not be able to go back and change your previous answers. Do you wish to continue?**

- Yes, I am ready to continue

|  |
| --- |

From here on, the questionnaire is about examinations for cell changes in the cervix (screening for cervical cancer).

Press “next” if you are ready to continue.

*(Below: non-numerical information module (provided to study arm 2, 3 and 4))*

**We are now going to give you some information about screening for cervical cancer.**

From the age of 23 to 49, you will be offered an examination for cell changes in the cervix every three years (screening for cervical cancer). You will be offered the examination whether you are vaccinated against HPV or not. You are now going to receive some information about this examination for cell changes.

Your general practitioner will take the sample during a gynecological examination. Cell changes can disappear by themselves, but in some cases they will develop into cervical cancer. If the changes are discovered and treated in time, you can avoid them developing into cervical cancer. However, it is not possible to know in advance which changes will disappear and which will develop into cancer in time. Therefore, doctors recommend a conisation if you have severe cell changes. During a conisation the doctor removes a cone-shaped piece of the cervix around the canal in the cervix.

1-8 out of 100 women will have severe bleeding in relation to the conisation. There is a slightly increased risk of preterm labor if you become pregnant in the future after a conisation.

There is a risk of false answers when you are examined for cell changes. There are two possible kinds:

1. False negative answer (false sense of security): You are told that your cell sample is normal, when you actually have cell changes. Cell changes develop very slowly, so in most cases they will not have developed into cancer before they are discovered at the next screening.
2. False positive answer (false alarm): The sample reveals cell changes, but further examinations show that they will **not** develop into cervical cancer.

The beneficial effect of the screening programme is that you prevent some cases of cervical cancer, because the cell changes are treated before they develop into cancer.

The harmful effects are for example false alarms, which can cause worry and lead to conisation among women who would never had developed cervical cancer.

The HPV vaccine does not give full protection against cervical cancer.

*(End non-numerical information module (provided to study arm 2, 3 and 4))*

*(Below: Numerical information module (provided to study arm 3, HPV vaccinated women)*

Researchers believe that the HPV vaccine prevents approximately 70% of all cervical cancers. If the vaccine works like the researchers expect, this can be expected:

**Beneficial effect of the screening programme (examinations for cell changes):**

Out of **1000 vaccinated women:**

7 out of 1000 women would have gotten cervical cancer before the age of 75 years **without** the screening programme.

3 out of 1000 women will get cervical cancer before the age of 75 years **with** the screening programme.

That means that 4 cases of cervical cancer per 1000 women are avoided because of the screening.

**Harmful effect of the screening programme (examinations for cell changes):**

Out of **1000 vaccinated women** who follow the entire screening programme:

440 will be called for further examinations at some point.

363 of these 440 will receive a false positive answer (false alarm).

44 of the 77 who are left will be treated with conisation right away.

33 of the 77 will have more examinations done, which in some cases will end with a conisation.

That means that at least 40 of the women that have a conisation never would have developed cervical cancer.

*(End numerical information module (provided to study arm 3, HPV vaccinated women)*

*(Below: Numerical information module (provided to: study arm 3, non-vaccinated women and study arm 4, first part,))*

**Beneficial effect of the screening programme (examinations for cell changes):**

Out of **1000 non-vaccinated women:**

22 out of 1000 women would have gotten cervical cancer before the age of 75 years **without** the screening programme.

9 out of 1000 women will get cervical cancer before the age of 75 years **with** the screening programme.

That means 13 cases of cervical cancer per 1000 women are avoided because of the screening.

**Harmful effect of the screening programme (examinations for cell changes):**

Out of **1000 non-vaccinated women** who follow the entire screening programme:

660 will be called for further examinations at some point.

506 of these 660 will receive a false positive answer (false alarm).

99 of the 154 who are left will be treated with conisation right away.

55 of the 154 will have more examinations done, which in some cases will end with a conisation.

That means at least 86 of the women that have a conisation never would have developed cervical cancer.

*(End numerical information module (provided to: study arm 3, non-vaccinated women and study arm 4, first part,))*

| *(Below: Numerical information module, provided to study arm 4, second part)*  Researchers believe that the HPV vaccine prevents approximately 70% of all cervical cancer cases. That means the conditions for cervical cancer screening will change compared to what we have so far told you. Below is a comparison of beneficial and harmful effects of the screening before and after the HPV vaccine:   \|  \| **Beneficial effect**  Per 1000 women \| **Harmful effect**  Per 1000 women who follow the entire screening programme \| \| --- \| --- \| --- \| \| **Without the HPV vaccine** \| 22 would have gotten cervical cancer **without** the screening programme.  9 will get cervical cancer **with** the screening programme.  **13 cases of cervical cancer are prevented because of the screening.** \| 660 will be called for further examinations at some point.  Out of these:  - 506 will get a false alarm  - 99 will be treated with conisation right away  - 86 or more will have an unnecessary conisation \| \| **With the HPV vaccine** \| 7 would have gotten cervical cancer **without** the screening programme.  3 will get cervical cancer **with** the screening programme.  **4 cases of cervical cancer are prevented because of the screening.** \| 440 will be called for further examinations at some point.  Out of these:  - 363 will get a false alarm  - 44 will be treated with conisation right away  - 40 or more will have an unnecessary conisation \|   *(End numerical information module, provided to study arm 4, second part)*  **When you turn 23 years, you will be offered a gynecological examination by your general practitioner. The doctor will take a sample of cells from the cervix (cervical cancer screening). The purpose of this sample is to check for cell changes, which may develop into cervical cancer. Do you think that you will say yes to have this examination for cell changes?**   - Yes - No - I do not know   **How certain are you of your answer to the previous question?**   - Certain - A little unsure - Unsure - I do not know   **Is your answer based on the information that you have been given in this questionnaire?**   - Yes, based on that information alone - Yes, to a high degree - Yes, to some degree - No, not at all - I do not know   *(Question below only provided to respondents that have answered “yes” to previos question concerning intended participation in screening)*  **For what reasons did you answer yes to have the examination for cell changes? (You are allowed to tick more than one box)** |
| --- | --- | --- | --- | --- | --- | --- | --- | --- | --- |

- I have been worried about getting cervical cancer for a while.
- This questionnaire has made me worried about getting cervical cancer.
- I always want more knowledge about my health.
- I hope that the cell sample can lower my risk of getting cervical cancer.
- I would regret to have declined if I later got cervical cancer
- When I receive an offer from the health care system, it is natural for me to accept it. I do not think much about beneficial and harmful effects of having the cell sample.
- There have been cases of cancer among my family or friends.
- There have been cases of cell changes among my family or friends.
- Other reasons
- I do not know

*(The three questions below are provided only to HPV vaccinated women in study arm 4 after the second part of the information module)*

**Do you think that you will say yes to have the examination for cell changes given the new information that you have just had?**

- Yes
- No
- I do not know

**How certain are you of your answer to the previous question?**

- Certain
- A little unsure
- Unsure
- I do not know

| **Is your answer based on the information that you have been given in this questionnaire and the table that you have just seen?**   - Yes, based on that information alone - Yes, to a high degree - Yes, to some degree - No, not at all - I do not know |
| --- |

Thank you very much for completing the questionnaire!
