# Supplementary material for: How does HPV vaccination status relate to risk perceptions and intention to participate in cervical screening? a survey study
Source: BMC Public Health. 2016 Aug 3;16:708. doi: 10.1186/s12889-016-3397-y (PMC4973036; doi:10.1186/s12889-016-3397-y)
Supplement: Additional file 2: Table S1. — Perceived cervical cancer risk as predictor of screening intention. (DOCX 14 kb) [file 12889_2016_3397_MOESM2_ESM.docx]

Table S1: Perceived cervical cancer risk as predictor of screening intention

|  | Total included in analysis  (unadjusted/  adjusted) | OR for yes to screening when perceived cervical cancer risk <11 per 1000 women (95% CI) | p-value | Adjusted OR for yes to screening when perceived cervical cancer risk <11 per 1000 women^*^  (95% CI) | p-value | OR for yes to screening when perceived cervical cancer risk <101 per 1000 women (95% CI) | p-value | Adjusted OR for yes to screening when perceived cervical cancer risk <101 per 1000 women^*^ (95% CI) | p-value | OR for yes to screening when perceived cervical cancer risk <501 per 1000 women (95% CI) | p-value | Adjusted OR for yes to screening when perceived cervical cancer risk <501 per 1000 women^*^ (95% CI) | p-value |
| --- | --- | --- | --- | --- | --- | --- | --- | --- | --- | --- | --- | --- | --- |
| **HPV vaccinated women** |  |  |  |  |  |  |  |  |  |  |  |  |  |
| Yes to screening | 645/615 | 1.13 (0.63 – 2.01) | 0.6918 | 1.28 (0.67 – 2.43) | 0.4549 | 1.03 (0.63 – 1.67) | 0.9145 | 0.94 (0.56 – 1.58) | 0.8088 | 0.49 (0.11 – 2.10) | 0.3356 | 0.45 (0.10 – 1.97) | 0.2856 |
| **Non-vaccinated women** |  |  |  |  |  |  |  |  |  |  |  |  |  |
| Yes to screening | 190/168 | 0.46 (0.12 – 1.77) | 0.2582 | 0.21 (0.04 – 1.09) | 0.0635 | 1.14 (0.59 – 2.20) | 0.6952 | 0.81 (0.36 – 1.82) | 0.6052 | 0.84 (0.41 – 1.73) | 0.6304 | 0.32 (0.12-0-86) | 0.0232 |

*Adjusted for study arm and the following socio-demographic variables: Year of birth, ethnicity, degree of urbanisation in area of habitat, completed educational level, parents’ educational level, and primary care contacts within the previous year.
